# Prolonged Gut Dysbiosis and Fecal Excretion of Hepatitis A Virus in Patients Infected with Human Immunodeficiency Virus

**DOI:** 10.3390/v13102101

**Published:** 2021-10-18

**Authors:** Aya Ishizaka, Michiko Koga, Taketoshi Mizutani, Lay Ahyoung Lim, Eisuke Adachi, Kazuhiko Ikeuchi, Ryuta Ueda, Haruyo Aoyagi, Satoshi Tanaka, Hiroshi Kiyono, Tetsuro Matano, Hideki Aizaki, Sachiyo Yoshio, Eiji Mita, Masamichi Muramatsu, Tatsuya Kanto, Takeya Tsutsumi, Hiroshi Yotsuyanagi

**Affiliations:** 1 Division of Infectious Diseases, Advanced Clinical Research Center, The Institute of Medical Science, The University of Tokyo, Tokyo 108-8639, Japan; ishizaka@ims.u-tokyo.ac.jp (A.I.); michiko@ims.u-tokyo.ac.jp (M.K.); tsutsumi@ims.u-tokyo.ac.jp (T.T.); 2 International Research and Development Center for Mucosal Vaccines, The Institute of Medical Science, The University of Tokyo, Tokyo 108-8639, Japan; kiyono@ims.u-tokyo.ac.jp; 3 Japan Foundation for AIDS Prevention, Tokyo 101-0064, Japan; 4 Department of Infectious Diseases and Applied Immunology, IMSUT Hospital of Institute of Medical Science, The University of Tokyo, Tokyo 108-8639, Japan; laylim@insti.kitasato-u.ac.jp (L.A.L.); e-adachi@ims.u-tokyo.ac.jp (E.A.); kikeuchi004@gmail.com (K.I.); 5 Department of Virology II, National Institute of Infectious Diseases, Tokyo 162-8640, Japan; ueda@niid.go.jp (R.U.); aoyagi@nih.go.jp (H.A.); aizaki@nih.go.jp (H.A.); muramatsu@nih.go.jp (M.M.); 6 Department of Gastroenterology and Hepatology, National Hospital Organization Osaka National Hospital, Osaka 540-0006, Japan; tanaka.satoshi.eg@mail.hosp.go.jp (S.T.); mita.eiji.zf@mail.hosp.go.jp (E.M.); 7 CU-UCSD Center for Mucosal Immunology, Allergy and Vaccines (cMAV), Department of Medicine, University of California San Diego, San Diego, CA 92093, USA; 8 AIDS Research Center, National Institute of Infectious Diseases, Tokyo 162-8640, Japan; tmatano@nih.go.jp; 9 Department of AIDS Vaccine Development, IMSUT Hospital, The Institute of Medical Science, The University of Tokyo, Tokyo 108-8639, Japan; 10 The Research Center for Hepatitis and Immunology, National Center for Global Health and Medicine, Chiba 272-8516, Japan; sachiyo@hospk.ncgm.go.jp (S.Y.); kantot@hospk.ncgm.go.jp (T.K.)

**Keywords:** HAV, HIV, microbiome, microbiome

## Abstract

Hepatitis A virus (HAV) causes transient acute infection, and little is known of viral shedding via the duodenum and into the intestinal environment, including the gut microbiome, from the period of infection until after the recovery of symptoms. Therefore, in this study, we aimed to comprehensively observe the amount of virus excreted into the intestinal tract, the changes in the intestinal microbiome, and the level of inflammation during the healing process. We used blood and stool specimens from patients with human immunodeficiency virus who were infected with HAV during the HAV outbreak in Japan in 2018. Moreover, we observed changes in fecal HAV RNA and quantified the plasma cytokine level and gut microbiome by 16S rRNA analysis from clinical onset to at least 6 months after healing. HAV was detected from clinical onset up to a period of more than 150 days. Immediately after infection, many pro-inflammatory cytokines were elicited, and some cytokines showed different behaviors. The intestinal microbiome changed significantly after infection (dysbiosis), and the dysbiosis continued for a long time after healing. These observations suggest that the immunocompromised state is associated with prolonged viral shedding into the intestinal tract and delayed recovery of the intestinal environment.

## 1. Introduction

Hepatitis A is one of the most common infectious liver diseases worldwide. It is caused by the hepatitis A virus (HAV), a positive-stranded RNA virus belonging to the genus Hepatovirus in the Picornaviridae family [1]. Hepatitis A is generally an acute and self-limiting hepatitis disease that rarely causes fulminant hepatitis. The main symptoms of this infection are fever, nausea, and jaundice with acute liver failure. Hepatitis A virus infections are common in developing countries, but sporadic outbreaks have been reported in developed countries [2]. The transmission of HAV mainly occurs via the fecal–oral route through water and food products contaminated with feces. In addition, sexual transmission is a well-recognized mode of HAV transmission, and men who have sex with men (MSM) are known to be at high risk of infection [3,4]. In 2018, an outbreak of hepatitis A was reported in a MSM population in Japan [5,6]. The viral strain in this outbreak was identified as the genotype 1A strain RIVM-HAV16-090, which was involved in a global outbreak in the MSM community in 2016–2017 [5,6].

After ingestion by the fecal-oral route, HAV spreads from the intestine to the liver via portal circulation. Viral particles of HAV that have replicated in the hepatocytes are discharged into the bile and excreted back into the duodenum [7,8]. Previous studies have shown that two different forms of infectious particles are found in HAV-infected individuals. Hepatitis A virus particles in the blood are quasi-enveloped (eHAV), encased in lipid bilayers derived from host cells, and are known to be resistant to neutralizing antibodies and to promote viral replication in liver tissue [9]. On the other hand, virions without envelopes are shed in the feces and remain stable in that environment for a long time, facilitating infection of new hosts [10,11]. In early studies, fecal HAV was revealed to be in complex with a specific immunoglobulin A [12,13]. There is some evidence that these immunocomplexes are transmitted to the liver via intestinal transcytosis and enhance reinfection of the liver [14,15]. However, it is unclear how long the infectivity of a virus that is shed via the intestinal tract actually persists.

Previous reports have shown that HAV RNA can be detected in both blood and feces for a long period of time in immunocompetent patients, even after alanine aminotransferase (ALT) levels have fallen into the normal range [16,17,18]. Viral shedding in feces occurs from 1 to 2 weeks prior to clinical onset and lasts up to 3 months or longer in immunocompetent patients, while plasma viremia was shown to be detectable for a median period of 42 days [16,17].

In recent years, it has become clear that the gut microbiome plays a role in the progression of liver diseases. Although hundreds of species of commensal bacteria colonize the human gut and have been shown to be deeply involved in liver function, little is known of the profile of the gut microbiome during HAV infection [19]. On the other hand, it has become clear that HIV-positive individuals have an unique gut microbiome profile. In our 16S rRNA metagenomic analysis of HIV patients with MSM, we observed that despite long-term viral suppression, the gut microbiome of HIV-infected patients is more aerobic than healthy individuals [20]. The altered gut microbiome in patients with HIV is considered to be influenced by the HIV infection itself, as well as the antiretroviral therapy (ART) regimen [21,22,23]. Recently, the unique pattern of gut microbiome related to sexual preference has also been well documented [24,25]. Although MSM are considered a high-risk group for HAV and HIV infection, there is limited information on the enterohepatic circulation of HAV when HAV is co-infected in HIV-infected individuals. Furthermore, the correlation between the gut microbiome and the immune dynamics of co-infection such as HAV infection in these patients is not fully understood. In this study, we comprehensively analyzed the correlation between changes in the gut microbiome due to acute HAV infection, and viral shedding and inflammation in the intestinal tract of HIV-infected individuals in a hepatitis A outbreak in the Japanese MSM-community in 2018.

## 2. Materials and Methods

### 2.1. Study Subjects and Sample Collection

A total of 10 patients with chronic HIV infection who were diagnosed with acute hepatitis A, 25 patients with chronic HIV infection who were without acute Hepatitis A, as well as 22 uninfected controls were enrolled in this study. Among the HIV positive patients, all of the patients were MSM and had been treated with antiretroviral therapy (ART) for more than 22 months. None of the patients with HAV took proton pump inhibitors. For clinical sample collection, among the 10 patients with HAV infection, 7 patients provided stool samples continuously for 68 to 225 days. Blood samples were obtained from 7 patients, including 3 patients who provided consent for cytokine measurement. None of the participants took antibiotics within the previous 2 weeks from stool collection.

### 2.2. Measuring HAV RNA Load in Fecal Samples

Patient feces/PBS (-) suspension (10%) was prepared, vigorously agitated, and centrifuged at 12,000 rpm for 20 min. The centrifuged supernatant was used for RNA purification, and RNA was purified according to the protocol of QIAamp Viral RNA Mini kit (Qiagen, Valencia, CA, USA), followed by DNase treatment using DNase I (Takara Bio), and extracted RNA was used as a template for RT-PCR. The cDNA was synthesized by SuperScript II RNase H- Reverse Transcriptase (Invitrogen, Carlsbad, CA, USA) according to the protocol, and then quantized using TaqMan Universal PCR Master mix with ABI PRISM 7700 system (Applied Biosystems, Foster City, CA, USA). The PCR condition was as follows: 50° C for 2 min, 95° C for 10 min once, then 95° C for 15 s, 56 °C for 1 min 45 times. Forward primer: 5′-AGG GTA ACA GCG GCG GAT AT-3′ (nt 449 to 468); reverse primer: 5′-ACA GCC CTG ACA RTC AAT YCA CT-3′ (nt 557 to 535); and TaqMan Probe: 5′-FAM-AGA CAA AAA CCA TTC AAC RCC GRA GGA C-TAMRA-3′ (nt 482 to 509). The primer location was based on the HAV strain, AH1/Japan (GenBank accession no. AB020564).

### 2.3. DNA Extraction

Stool samples were washed three times in a SM-plus buffer (100 mM NaCl, 50 mM Tris-HCl [pH 7.4], 8 mM MgSO_4_, 5 mM CaCl_2_, and 0.01% gelatin) and centrifuged to remove the supernatant. The pellet was resuspended in a SM-plus buffer and large debris were removed by filtering the stool suspension through a 100 μm cell strainer. The resulting suspension was incubated with 20 mM ethylenediaminetetraacetic acid (EDTA), 500 U/mL achromopeptidase (Sigma-Aldrich, St. Louis, MO, USA), and 0.1 mg/mL human lysozyme (Sigma-Aldrich) at 37 °C for 1 h, followed by overnight incubation with 50 μg/mL protease K (Nacalai Tesque, Kyoto, Japan) and 0.05% sodium dodecyl sulfate at 37 °C. Bacterial DNA was extracted using the phenol-chloroform method and purified with a QIAquick PCR Purification Kit (Qiagen).

### 2.4. Construction of DNA Library and Sequencing

The 16S V3 and V4 regions were amplified using the KAPA SYBR Fast qPCR Kit (Kapa Biosystems, Wilmington, MA, USA). The primary PCR protocol comprised an initialization step at 94 °C for 2 min and 20 cycles at 98 °C for 10 s and 68 °C for 15 s using the following primer pair: Forward primer, 5′-ACA CGA CGC TCT TCC GAT CTC CTA CGG GNG GCW GCA G-3′ and reverse primer, 5′-GAC GTG TGC TCT TCC GAT CTG ACT ACH VGG GTA TCT AAT CC-3′. The PCR amplicon was purified with Agencourt AMPure XP magnetic beads (Beckman Coulter) per the manufacturer’s instructions. The secondary PCR protocol consisted of an initialization step at 94 °C for 45 s, and 8 cycles at 98 °C for 15 s, 50 °C for 30 s, and 72 °C for 30 s using NEB Next Multiplex Oligos for Illumina (Dual Index Primers Set 1; New England Biolabs [NEB], Ipswich, MA, USA). The final PCR amplicon was purified with Agencourt AMPure XP magnetic beads and verified using agarose gel electrophoresis. The samples were normalized and pooled, followed by sequencing on the MiSeq platform using the MiSeq Reagent Kit V3 (Illumina, San Diego, CA, USA).

### 2.5. Sequence Data Analyses

The 16S rRNA reads were analyzed using the Quantitative Insights Into Microbial Ecology version 2 (QIIME2) pipeline version 2020.6 [26]. Paired-end reads were merged and denoised using DADA2 [27]. Sequences were clustered into operational taxonomic units (OTUs) at a threshold of 97% sequence identity against the Silva reference database version 138 [28].

### 2.6. Measurement of the Cytokine Profile

Blood samples were obtained from patients with HAV infection. Plasma cytokine levels were quantified using a Bio-Plex System (Bio-Rad Laboratories, Hercules, CA, USA). The measurement was performed using multiplex assay kits, Bio-Plex Pro Human Chemokine Panel, 40-Plex #171AK99MR2, and Bio-Plex Pro Human Inflammation Panel 1, 37-Plex #171AL001M in accordance with the manufacturer’s specifications.

### 2.7. RNA Purification and qRT-PCR Analysis

Short Intracellular HIV RNA was measured by a method previously described [21]. For the analysis of the RNA, a portion of small RNA (<200 nt) from patient blood-derived PBMC was purified using Isogen II (Nippon Gene, Toyama, Japan). The cDNA was synthesized using a 5 × miScript HiFlex Buffer (Qiagen, Valencia, CA) supplied with the miScript II RT kit. Amplification reactions were performed on a CFX96 real-time PCR detection system (Bio-Rad Laboratories) using Premix Ex Taq [Probe qPCR] (TaKaRa Bio). The amount of RNA copies was calculated per 10^6^ peripheral blood mononuclear cells (PBMCs).

## 3. Results

### 3.1. General Characteristics of Participants

During the period from June 2018 to March 2020, a total of 10 patients with chronic HIV infection were diagnosed as having acute HAV infection. All of the patients were male and MSM. The median (interquartile range) age was 46 (36.8–52.3) years for the patients with HIV and acute HAV infection, 47 (42–50.5) years for the 25 HIV patients in the comparison group, and 45 (34–50.3) years for the 22 healthy controls. All of the patients were on ART, the CD4 count according to the most recent test before HAV infection was 579 (483–707.5) cells/μL, and the HIV viral load was under the detection limit (Table 1). Detailed clinical information for 10 patients is given in Table 2 and Table 3. Five of these patients had underlying diseases: Insomnia, atopy, hypertension, and dyslipidemia (Table 2). In Table 3, the values of liver markers (AST, ALT, and T-Bil) and antibody levels (HA-IgM) during HAV infection were shown.

### 3.2. Time Course of Viral Shedding and Clinical Markers during HAV Co-Infection among Patients with Chronic HIV Infection

**Figure 1:**
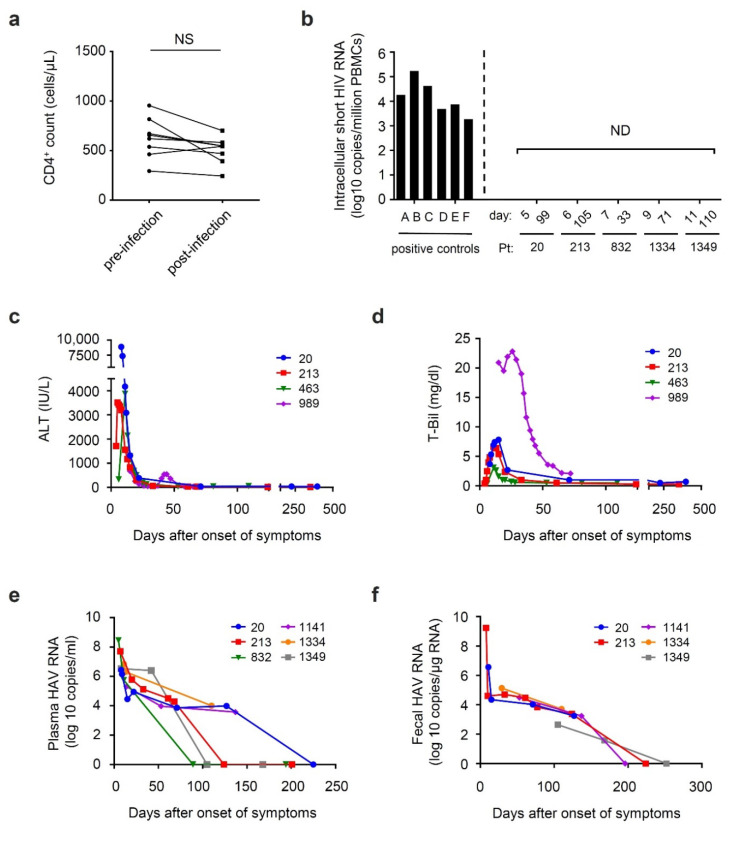
Changes in the levels of human immunodeficiency virus (HIV) in the blood and hepatitis A virus (HAV) excretion in the stool during the period from immediately after acute HAV infection to after healing. The day of clinical onset was set to day 1 on the horizontal axis; (**a**) CD4 count (cells/mL) of patients with HIV after HAV infection; (**b**) intracellular short HIV transcripts in peripheral blood mononuclear cells (PBMCs) (copies/million PBMCs). Patients A-F shown on the left were positive controls for the transcripts; (**c**) the levels of alanine aminotransferase (ALT) in plasma fractions of patients (U/mL); (**d**) the levels of total bilirubin (T-Bil) in plasma fraction of patients (mg/dL); (**e**,**f**) the levels of HAV-RNA in the plasma (copies/mL); (**e**) and the feces (copies/μg RNA) (**f**) of patients. NS: Not significant; ND: Not detected.

During the course of HAV infection, the CD4 count and HIV viral load were not affected by the acute HAV infection (Figure 1a, and data not shown). Intracellular short HIV RNA, which reflects persistent transactivation of the HIV promoter and well-correlates with the levels of CD8^+^ T-cell activation in aviremic patients with long-term successful ART [29], was not detected as well (Figure 1b). Levels of liver function marker, such as the levels of ALT and total bilirubin (T-Bil) were shown in Figure 1c,d. The ALT levels were observed to be the highest at clinical onset of HAV infection, while the levels of T-Bil peak at several days after onset. The values of those markers rapidly decreased over time, reaching normal levels around 30 days after onset in all of the patients, except for patient no. 989, whose ALT and T-Bil levels were observed to rebound (Figure 1c,d). The HAV viral load was at the highest titer level in the first point sample of each patient and gradually decreased over time (Figure 1e). Among these patients, three patients (no. 20, 1141, and 1334) and one patient (no. 213) were positive for HAV RNA for over 100 days and 50 days after clinical onset, respectively. As for fecal excretion of HAV RNA, all of the patients whose feces were continuously collected were positive for HAV RNA for 100 days after clinical onset (Figure 1f). Among these, patient no. 1349 was positive for HAV RNA in his feces at day 168.

### 3.3. Time Course of Chemokine Expression during Acute HAV Infection

**Figure 2:**
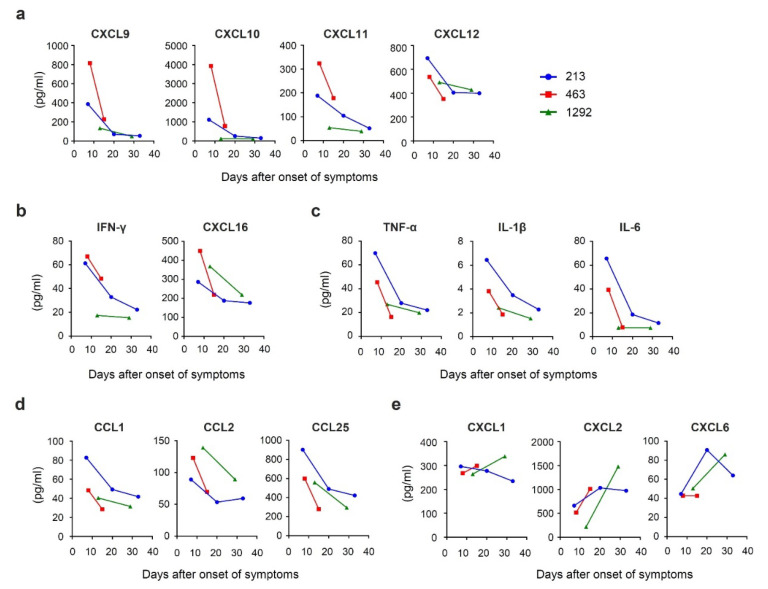
Changes of cytokine profile after clinical onset of HAV infection. Measurements of (**a**) CXCL9, CXCL10, CXCL11, and CXCL12; (**b**) IFN-γ and CXCL16; (**c**) tumor necrosis factor (TNF)-α, interleukin (IL)-1β, and IL-6; (**d**) CCL1, CCl2, and CCL25; and (**e**) CXCL1, CXCL2, and CXCL6 in serum of patients. The clinical onset was set to day 1.

Several chemokines, such as CXCL9, CXCL10, CXCL11, and CXCL12 were previously described as being involved in liver diseases [30,31,32,33]. These chemokines were observed to be present in high levels at clinical onset and gradually decreased over a number of days in two of the patients, although this could not be fully determined in Patient no. 463 (Figure 2a). Among these chemokines, CXCL9, CXCL10, and CXCL11 are known as interferon gamma (IFN-γ)-induced chemokines and are involved in Th1 immune response [34]. Similar secretion kinetics were observed for IFN-γ itself and for CXCL16 (Figure 2b), which has been known to induce migration of natural killer T (NKT) cells, one of the most efficient producers of IFN-γ [35]. In addition, a similar pattern of change was observed in several pro-inflammatory cytokines, such as tumor necrosis factor (TNF)-α, interleukin (IL)-1β, and IL-6 (Figure 2c), as well as chemokines that induce migration of monocytes and macrophages, such as CCL1, CCL2, and CCL25 (Figure 2d). The decrease in the level of these chemokines was similar to ALT, as shown in Figure 1d. On the other hand, some chemokines did not decrease, such as CXCL1, CXCL2, and CXCL6, which are reported to bind to CXCR2 receptors and mediate neutrophil trafficking [36], showing sustained or elevated secretion kinetics (Figure 2e).

### 3.4. Prolonged Fecal Dysbiosis after HAV Infection

**Figure 3:**
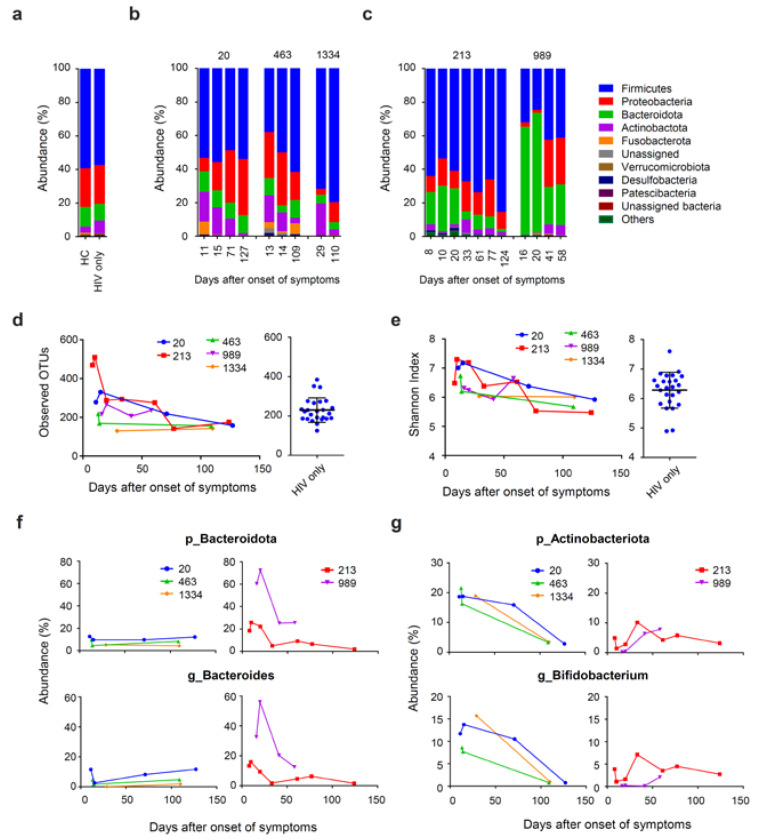
16S rRNA analysis of changes in the intestinal microbiome of patients after hepatitis A virus (HAV) infection. (**a**–**e**) Bacterial taxa profile (Phyla) in the gut microbiome of each patient: (**a**) Average of bacterial taxa profile (Phyla) of healthy cohorts (HC), patients with HIV (not with HAV); (**b**,**c**) the taxa profile (Phyla); (**d**) observed operational taxonomic units (OTUs); (**e**) Shannon index of the gut microbiome of individual patients; and (**f**,**g**) bacterial taxa profile (Phyla and genus) in the gut microbiome of each patient. The clinical onset was set to day 1. Due to the small number of analyses, statistically significant differences in changes in the gut microbiota could not be confirmed.

To monitor the impact of HAV infection on the gut microbiome, we performed 16S ribosomal RNA sequencing and investigated the microbial distributions at the phylum level. The dominant phyla in the gut microbe were Firmicutes, Proteobacteria, Bacteroidetes, and Actinobacteria in both patients with chronic HIV infection and healthy controls (Figure 3a). We monitored the sequential changes in the gut microbiome during acute HAV infection and after recovery from infection. We observed the temporary enrichment of low abundance phyla at clinical onset, such as the phyla Fusobacteria and Desulfobacterota (Figure 3b,c). This result was consistent with the observation of a transient increase and subsequent decrease in the alpha-diversity of these patients. Alpha diversity determined by the observed OTUs and Shannon index values were higher in patients with HAV after clinical onset compared to those of patients without HAV (Figure 3d,e). Both observed OTUs and Shannon index values were gradually decreased over a duration of 100 days to a degree similar to those of patients without HAV. In addition, among three out of the five patients we analyzed, we observed enrichment in Actinobacteria and lower abundance of Proteobacteria compared to patients without HAV (Figure 3b). In the other two patients, we observed enrichment of Bacteroidota and lower abundance of Proteobacteria (Figure 3c). The composition of microbiome at the phylum level became similar to patients without HAV over a duration of about 100 days (Figure 3b,c). Within the phyla Actinobacteria and Bacteroidota, the genera *Bifidobacterium* and *Bacteroides*, respectively were the taxa majorly responsible for enrichment of these phyla, respectively (Figure 3f,g). Together, the impact of HAV infection on the gut microbiome continued not only during acute HAV infection, but also for a long time after recovery of clinical symptoms.

## 4. Discussion

Prior to the current study, only two published studies had followed the duration of viremia and fecal excretion of HAV RNA after HAV infection in HIV patients. One analysis compared 15 HIV-1-infected MSM with an equal number of age-matched non-HIV-infected patients and reported that the HAV load was higher in HIV-1-infected patients than in non-infected patients. In addition, the duration of viremia in HIV-1-infected patients (median 53 days) was significantly longer than in non-infected patients (median 22 days), suggesting that HIV-1 infection is associated with prolonged HAV viremia [37]. The other study, albeit a small analysis of only one patient, reported that a patient with HIV who was ART-naïve and suffering from acute HAV infection exhibited prolonged plasma viremia for up to 256 days and Hepatitis A virus RNA was detected from feces at day 106 after the onset of symptoms [18]. In the current study, we observed that HAV viremia lasted for over 100 days after the onset of symptoms in three out of six patients with HIV. In addition, fecal shedding of HAV RNA in these patients lasted for more than 100 days. Although all the patients were on ART and achieved virologic suppression, this was clearly longer than the immunocompetent patients, which was previously reported as a median period of 42 days for HAV viremia and 81 days for fecal excretion [14]. In consideration of previous studies showing that CD4^+^ T cells play an important role in terminating HAV infection in a chimpanzee model [38], our observations on HAV viremia fecal shedding of HAV RNA suggest that HIV-infected patients need a longer period of time to eliminate HAV from their bodies. This may be due to the chronic immune exhaustion that has been reported in HIV patients [39].

We observed that proinflammatory cytokines, as well as chemokines involved in the cellular immune response were of high levels at clinical onset and decreased over time. Reduction of these cytokines and chemokines occurred at the same timing as the decrease in markers of liver damage. This is in line with earlier studies, showing that the host immune response, such as IFN-γ-producing cytotoxic CD8^+^ T cells, contributes to liver damage during acute HAV infection [40,41]. In addition, we observed no effect of acute HAV infection on CD4 counts, plasma HIV viral load, as well as cellular HIV RNA, which was consistent with the study on the ART-naïve patient with HAV infection [18]. This may be due to the fact that the acute HAV infection induces only a limited type of I IFN response [42]. It is known that HAV blocks the innate immune signaling pathway via digesting key proteins of the signaling pathway that induce the type I INF synthesis, such as TIR-domain-containing adapter-inducing interferon-β (TRIF) [43], mitochondrial antiviral signaling (MAVS) protein [44], and NF-kappa-B essential modulator (NEMO) [45]. In chimpanzee models, type I IFN is induced in a very early stage of HAV infection, but it is rapidly downregulated when the level of HAV RNA increases in the liver [46]. The attenuation of IFN induction in the early stages of infection may result in the limited activation of CD4^+^ T cells that harbor HIV proviruses, and is thus less likely to lead to the transactivation of HIV promoter. On the other hand, the secretion levels of chemokines CXCL1, CXCL2, and CXCL6 did not decrease even after ALT reached normal levels. This may be due to HAV shedding and gut dysbiosis that continues even after the ALT level has decreased to the normal range. Previous reports have shown that CXCL1, 2, and 6 have a function in the chemotaxis of neutrophils to sites of inflammation [36]. This observation suggests that the immune response continues consistently even after the acute infection has subsided. The effect of HIV infection on the clinical course of hepatitis A needs to be further evaluated by comparing these patients with patients infected with HAV without HIV infection.

Earlier studies have revealed the association between the gut dysbiosis and different chronic liver diseases, such as chronic infection with hepatitis B virus (HBV) or hepatitis C virus (HCV), as well as nonalcoholic fatty liver disease. However, to the best of our knowledge, there have been no reports of gut microbiome analysis in patients with acute HAV. This may due to the fact that hepatitis A is an acute, self-limiting infection, and there is difficulty in collecting clinical samples serially for an extended period, including the period of post-recovery of symptoms. However, given that the gut microbiome is closely related with bile acid metabolism and circulation, it is important to clarify the gut dysbiosis in acute hepatitis to better understand the pathogenesis of the disease. In chronic liver diseases, the relative composition of gut microbiome is reported to be changed by the increasing proportion of facultative anaerobic bacteria, as well as the decreasing proportion of strictly anaerobic bacteria. For example, depletion of *Bifidobacterium* and *Bacteroides,* as well as enrichment of *Proteobacteria* were reported in patients with chronic HBV infection and those with cirrhosis [47,48,49]. In chronic HCV infection, an increasing abundance of *Prevotella*, *Faecalibacterium*, *Acinetobacter*, *Veillonella*, as well as *Phascolarctobacterium* was reported, and gut dysbiosis was linked to the progression of the disease [50]. However, the gut dysbiosis we found in patients with acute hepatitis A was different from that found in patients with chronic liver disease. The difference in dysbiosis may be due to the different effects of chronic inflammation associated with chronic liver disease and acute liver injury. One explanation for this is the fact that we have observed a transient increase in OTU immediately after the infection (Figure 3d). The cause may be due to direct effects, such as physical contact between the virus and intestinal bacteria, in addition to the effects of inflammation during acute infection when a large amount of virus is discharged into the intestinal tract. Given that bile has a function of neutralizing stomach acid, the rapidly decreased liver function due to the HAV infection can result in lower pH in the gut. The intestinal pH can greatly influence the microbial composition in the gut [51]. A previous study on the impact of mildly acidic pH on the growth of different species of fecal bacteria showed that the species within *Bifidobacterium* grew well, but Proteobacteria grew poorly at pH 5.5 than at pH 6.7 [52]. We observed a temporary reduction of the phylum Proteobacteria, as well as an increase of the genera *Bifidobacterium* in three out of five patients with HIV and acute HAV infection. The peak of gut dysbiosis that we observed was about 2 to 4 weeks after clinical onset, which succeeds the peak of ALT and T-Bil. Therefore, this gut dysbiosis may reflect a decrease in the pH of the intestines. In addition, previous reports revealed that specific bacteria are involved in the metabolism of specific types of bile acids [53,54]. Taken together, the interaction between the abnormal bile secretion and alteration of gut microbiome may be one of the reasons why gut dysbiosis continues in patients with acute HAV infection, even after recovery from the clinical symptoms.

One of the limitations of this study was the evaluation of limited samples, which may introduce bias owing to individual differences and the impact of the ART regimen. To date, individual and combinations of antiretroviral drugs have been reported to affect the gut microbiome [23]. For example, a protease inhibitor-based regimen was reported to be associated more with microbial translocation and gut endothelial damage as compared with a non-nucleoside reverse transcriptase inhibitors-based regimen [55]. To date, there are few studies on ARVs and the gut microbiome, and little is known on this issue. However, the results of this study, together with previous reports, suggest that HIV-infected individuals shed HAV virus in their feces for a long period of time. Furthermore, this study shows that the intestinal microbiome of HIV-infected individuals changes significantly during the acute phase of HAV infection, and that recovery takes time even after the symptoms have subsided. For a more detailed understanding, it would be important to make a direct comparison between the HAV patients with and without HIV in HAV viral load in blood and feces, as well as the HAV-related changes in the gut microbiota. The role of enteric bacteria in the enterohepatic circulation of HAV is currently unknown, but it may lead to important insights into understanding the life cycle of HAV. In the future, it is expected that the improved understanding of the intestinal environment, including the gut microbiome, will lead to a better understanding of the pathogenesis of hepatitis A and the interaction between the host and the pathogen.

## Figures and Tables

**Table 1:** Comparison of the characteristics among HIV patients with or without hepatitis A and healthy controls.

	HIV and Acute HAV	HIV Alone	Healthy Controls
Total patients (No.)	10	25	22
Males (No.)	10 (100%)	25 (100%)	22 (100%)
MSM (No.)	10 (100%)	24 (96%)	-
Age in years	46 (36.8–52.3)	47 (42–50.5)	45 (34–50.3)
BMI	23.6 (20.8–25.2)	23.6 (20.8–26.1)	-
HIV Viral load < 20 copies/mL (No.)	10 (100%) ^1^	25 (100%)	-
CD4 count (cells/ul)	579 (483–707.5) ^1^	613 (517–731.5)	-
Duration of ART (years)	8 (3.5–14)	8 (6.5–13)	-
Peak serum ALT (IU/L)	3540 (581.3–4001.3)	-	-
Peak serum AST (IU/L)	1958 (1288–3673.8)		
Peak serum T-Bil (mg/dL)	6.4 (3.0–11.8)	-	-

Data are shown as the median and interquartile range unless otherwise described. ^1^ Prior to HAV infection. BMI: Body mass index; AST: Aspartate aminotransferase; -: not applicable.

**Table 2:** Basic characteristics of HIV patients with hepatitis A.

Patient No.	Age	BMI	Years after HIV Diagnosis	CD4 Counts (/μL) ^1^	CD8 Counts (/μL) ^1^	CD4/CD8 Ratio ^1^	HIV-RNA Load (Copies/mL) ^1^	ART Regimen	Underlying Health Conditions
20	40	24.2	9	508	656	0.8	<20	TAF/FTC RPV	Dyslipidemia
213	56	24.8	24	538	1035	0.5	<20	TDF/FTC DTG	Insomnia
463	49	27.7	20	955	1124	0.8	<20	ABC/3TC/DTG	Hypertension, dyslipidemia
708	44	23.1	12	490	378	1.3	<20	ABC/3TC DRV/c	-
832	53	24.2	7	817	839	1	<20	TAF/FTC DTG	-
989	36	21.3	10	620	589	1.1	<20	TAF/FTC/RPV	Dyslipidemia
1141	48	26.4	6	671	447	1.5	<20	ABC/3TC RAL	-
1292	52	19.2	4	293	605	0.5	<20	TAF/FTC DTG	-
1334	27	17.7	2	656	525	1.2	<20	TAF/FTC DTG	-
1349	37	22.0	1	462	539	0.9	<20	ABC/3TC/DTG	Atopic dermatitis

^1^ Prior to HAV infection. TAF: Tenofovir alafenamide fumarate; FTC: Emtricitabine; RPV: Rilpivirine; TDF: Tenofovir disoproxil fumarate; DTG: Dolutegravir; ABC: Abacavir; 3TC: Lamivudine; DRV/c: Darunavir/cobicistat; RAL: Raltegravir; -: no underlying health conditions.

**Table 3:** Laboratory data associated with hepatitis A.

Patient No.	Initial HA-IgM (s/co)	ALT (IU/L)		Max AST (IU/L)	Max T-Bil (mg/dL)
		Before	Max		
20	5.7	34	8877	6996	7.8
213	0.58	23	3515	3530	6.6
463	1.09	114	3880	1722	3.1
708	3.25	16	2589	2194	15.2
832	3.24	21	3985	4105	6.2
989	11.2	11	677	1400	22.8
1141	11.7	26	294	152	2.5
1292	2.04	10	174	952	2.2
1334	10.1	8	4050	2860	10.6
1349	11.6	22	3565	1663	4.5

## Data Availability

The data presented in this study are available on request from the corresponding authors.
